# A Diverse Repertoire of CD4 T Cells Targets the Immediate-Early 1 Protein of Human Cytomegalovirus

**DOI:** 10.3389/fimmu.2015.00598

**Published:** 2015-11-25

**Authors:** Stefanie Ameres, Xiaoling Liang, Martina Wiesner, Josef Mautner, Andreas Moosmann

**Affiliations:** ^1^Clinical Cooperation Group Immunooncology, Helmholtz Zentrum München and Ludwig-Maximilians-Universität, Munich, Germany; ^2^Research Group Host Control of Viral Latency and Reactivation, Helmholtz Zentrum München, Munich, Germany; ^3^German Research Center for Infection Research (DZIF), Munich, Germany; ^4^Clinical Cooperation Group Pediatric Tumor Immunology, Helmholtz Zentrum München and Technische Universität München, Munich, Germany

**Keywords:** *cytomegalovirus*, CMV, CD4 T cells, HLA class II, IE-1

## Abstract

T-cell responses to the immediate-early 1 (IE-1) protein of human cytomegalovirus (HCMV) are associated with protection from viral disease. Thus, IE-1 is a promising target for immunotherapy. CD8 T-cell responses to IE-1 are generally strong. In contrast, CD4 T-cell responses to IE-1 were described to be comparatively infrequent or undetectable in HCMV carriers, and information on their target epitopes and their function has been limited. To analyze the repertoire of IE-1-specific CD4 T cells, we expanded them from healthy donors with autologous IE-1-expressing mini-Epstein–Barr virus-transformed B-cell lines and established IE-1-specific CD4 T-cell clones. Clones from seven out of seven HCMV-positive donors recognized endogenously processed IE-1 epitopes restricted through HLA-DR, DQ, or DP. Three to seven IE-1 epitopes were recognized per donor. Cumulatively, about 27 different HLA/peptide class II complexes were recognized by 117 IE-1-specific clones. Our results suggest that a highly diversified repertoire of IE-1-specific CD4 T cells targeting multiple epitopes is usually present in healthy HCMV carriers. Therefore, multiepitope approaches to immunomonitoring and immunotherapy will make optimal use of this potentially important class of HCMV-specific effector cells.

## Introduction

Persistent infection with human cytomegalovirus (HCMV) is widespread in healthy humans (1). Control of viral replication and disease is believed to critically depend on HCMV-specific T cells (2). In transplanted patients whose virus-specific T-cell response is impaired, HCMV can cause severe and potentially fatal disease (3). Likewise, the danger of harmful congenital infection is increased when the mother acquires HCMV for the first time during pregnancy (4). Reconstitution of HCMV-specific T cells by adoptive transfer is associated with control of HCMV infection and disease, in particular in the situation after allogeneic stem cell transplantation where a compatible donor of HCMV-specific T cells is available (5). Although some approaches to adoptive T-cell transfer primarily aim at reconstituting HCMV-specific CD8 T cells (6, 7), it appears that HCMV-specific CD4 T cells play an important role in therapeutic success (5, 8).

Many HCMV antigens can be targets of virus-specific T cells (9). Two of these, the virion tegument phosphoprotein pp65 and the immediate-early protein IE-1, have been in the focus of *in vitro* studies as well as human T-cell therapy, since they are dominant targets of the virus-specific CD8 T-cell response (10, 11). Since IE proteins are the first to be expressed in viral reactivation and orchestrate subsequent virus replication, it appears plausible that IE antigen-specific T cells may be of particular importance in antiviral control. Accordingly, experiments with murine CMV have demonstrated that IE-specific CD8 T cells protect from disease (12, 13) and inhibit viral replication by sensing of reactivation in its first stage (14). Human T-cell responses to IE-1 have seemed more enigmatic. Compared with pp65-specific T cells, IE-1-specific CD8 T cells are associated with superior protection from viral disease in transplant patients (15, 16). Paradoxically, IE-1-specific CD8 T cells were found to recognize infected cells *in vitro* much less well than T cells specific for structural antigens, due to interference by HLA-modulating viral proteins (17). We recently resolved this apparent contradiction by showing that certain HLA allotypes are resistant to downregulation by viral immunoevasins and thus present IE-1 epitopes that are well recognized by strongly immunodominant populations of CD8 T cells (18, 19).

The general importance of IE-1-specific CD4 T cells has been unclear, in particular because of their relative rarity. Already in 1988, such T cells were identified in healthy HCMV carriers (20), and several HLA-DR-restricted T-cell epitopes of IE-1 have been characterized (21–23). However, employing different techniques to measure IE-1-specific CD4 T cells, several studies identified them in only about one-third of HCMV-positive donors (9, 22, 24), whereas others did not detect them at all (25, 26).

These findings raised the question of whether IE-1-specific CD4 T cells are a regular component of the HCMV-specific T-cell response and can be expected to mediate antiviral functions in a majority of healthy carriers or patients after successful immunotherapy. Therefore, we expanded such T cells from healthy HCMV-positive donors by stimulation with autologous B cells carrying a mini-Epstein–Barr virus (mini-EBV) expressing IE-1. We established a series of IE-1-specific CD4 T-cell clones and studied their epitope specificities and functions.

## Materials and Methods

### Ethics Statement and Donors

Mononuclear cells from standard blood donations by anonymous healthy adult donors were obtained from the Institute for Transfusion Medicine, University of Ulm, Germany. The institutional review board (Ethikkommission, Klinikum der Universität München, Grosshadern, Munich, Germany) approved this procedure. All work was conducted according to the principles expressed in the Helsinki Declaration.

HLA class II types of the seven donors who were analyzed for their IE-1-specific T-cell repertoire are provided in Table 1. High-resolution HLA typing was performed by PCR-based methods (IMGM, Martinsried, Germany). The HCMV IgG serostatus was determined (Max von Pettenkofer Institute, Munich, Germany).

**Table 1:** **HLA class II types of T-cell donors**.

Donor	HLA-DRB1	HLA-DRB3/4/5	HLA-DQA1	HLA-DQB1	HLA-DPA1	HLA-DPB1
ALT	*0401, *1501	4*0103, 5*0101	*0102, *0303	*0301, *0602	*0103, *0201	*0401, *1101
ARZ	*0403, *1501	4*0103, 5*0101	*0102, *0301	*0302, *0602	*0103, –	*0201, *0301
AJU	*0701, *1301	3*0202, 4*0101	*0103, *0201	*0202, *0603	*0103, –	*0401, *2001
F61	*0102, *0701	4*0101	*0101, *0201	*0202, *0501	*0103, *0201	*0402, *1401
F63	*0101, *0803	None	n.d.	*03, *05	n.d.	*0401, –
F64	*0701, *1301	3*0101, 4*0101	*0103, *0201	*0202, *0603	*0103, *0201	*0201, *1101
F65	*1454, *1501	3*0202, 5*0101	*0101, *0102	*0503, *0602	*0103, –	*0201, *0301

*n.d., not determined*.

### B Cells, Plasmids, and Peptides

Standard cell culture medium was RPMI1640 (Invitrogen) with 10% fetal calf serum (Invitrogen), penicillin (100 U/mL), streptomycin (100 g/mL, Invitrogen), and 100nM sodium selenite (ICN). Mini-lymphoblastoid cell lines (mini-LCLs) stably expressing HCMV IE-1 (18) were generated as described (27) by infection of PBMCs with packaged recombinant mini-EBV carrying the HCMV IE-1 gene from HCMV strain AD169 under the SV40 early promoter. Mini-LCLs lacking expression of a heterologous protein were analogously generated. CD40-stimulated B-cell cultures were established and maintained as described (28). DG75 cells were from ATCC. Sequences coding for HLA class II chains were amplified from PBMCs or LCLs and cloned into pCMVcyto. Peptides were synthesized to >70% purity by JPT (Berlin), resuspended in 100% dimethyl sulfoxide (DMSO), and stored at −20°C. To identify IE-1 peptides recognized by T cells, we used a peptide library of 120 peptides with a length of 15 amino acids and an overlap of 11 amino acids, which covered the entire 491-amino acid protein sequence of IE-1 from HCMV strain AD169. To facilitate screening, peptides were distributed to 12 “vertical” subpools (subpools 1–12) and 10 “horizontal” subpools (subpools 13–22), in analogy to the procedure described for pp65 (29). Subpool 23 contained nine additional 15-meric peptides from IE-1 that covered selected sequence variants appearing in HCMV strains Toledo and TB40. Protein sequences follow GenBank entries FJ527563 (AD169 substrain varUC, complete genome), GU937742 (strain Toledo, complete genome), and KF297339 (strain TB40/E clone Lisa, complete genome).

### T Cells

IE-1-specific polyclonal T-cell lines were prepared by restimulation of PBMCs from HCMV-seropositive donors with irradiated autologous IE-1-expressing mini-LCLs, as previously described for pp65 (30). Per well of a 24-well plate, 2 million PBMC and 50,000 irradiated mini-LCL (50 Gy) were cocultivated in 2 mL medium. On day 10 and then after intervals of 7 days, cells were pooled, counted, and replated at 1 million cells in 2 mL medium per well, adding freshly irradiated mini-LCL cells as stimulators at an effector–stimulator ratio of 4:1. Cells were re-fed or expanded at least once between stimulations. From day 15 onward, culture medium was supplemented with rIL-2 (50–100 U/mL; Proleukin, Novartis). After 6–8 weeks of cultivation, CD4 T cells were positively isolated using CD4 Microbeads (Miltenyi Biotec) and submitted to limiting dilution to obtain T-cell clones. For limiting dilution, T cells (0.7 or 2.5 cells/well) were seeded into round-bottom 96-well plates (200 μL/well) in medium supplemented with 1,000 U/mL rIL-2, 1 × 10^5^/mL irradiated (50 Gy) autologous IE-1-expressing mini-LCLs, and 1.5 × 10^6^/mL of a mixture of irradiated (50 Gy) allogeneic PBMCs from at least three different donors. Outgrowing T-cell clones were expanded in round-bottom 96-well plates by restimulating every 2 weeks under the same conditions. For analysis by flow cytometry, T cells were stained with CD4-FITC and CD8-APC (BioLegend) for 20 min on ice, washed with PBS + 2% FCS, and analyzed on a BD Biosciences FACSCalibur flow cytometer. Data analysis was performed using FlowJo 9.4.11 software (Tree Star).

### T-Cell Effector Assays

PBMCs were analyzed for specific IFN-γ secretion in ELISpot, T-cell clones in IFN-γ ELISA. Antigenic peptides were used at final concentrations of 5 μg/mL per peptide when using single peptides or subpools of up to 12 peptides, except when indicated otherwise. IFN-γ ELISpot analyses (Mabtech, Nacka, Sweden) were performed in 96-well MultiScreen HTC Filter Plates (Millipore). After antibody coating of the wells, 200,000 PBMCs were distributed to each well, directly loaded with antigenic peptide, and incubated in a total of 200 μL medium per well for 16–18 h at 37°C and 5% CO_2_. After counterstaining with biotinylated secondary antibody and streptavidin-AP, spots were developed using the AP Conjugate Substrate Kit from Bio-Rad and visually counted after scanning. For ELISPOT analysis of cultivated polyclonal T cells, 2,000–10,000 T cells were cocultivated with 50,000 CD40-stimulated B cells preloaded with peptides, and incubated and processed as above.

For initial screening of T-cell clones for IE-1 specificity, 10-μL aliquots of cloning wells (containing approximately 2,000–10,000 T cells) were coincubated with autologous ctrl-mini-LCL, IE-1-mini-LCL, and CD40-stimulated B cells (50,000 cells/well) in 200 μL/well in V-bottom 96-well plates at 37°C and 5% CO_2_ overnight, and supernatants were analyzed in IFN-γ ELISA (Mabtech, Nacka, Sweden). For determination of peptide specificity, T cells (10,000 cells/well) were incubated overnight with CD40-stimulated B cells (20,000 cells/well) in the presence of peptides (5 μg/mL per peptide) and analyzed in IFN-γ ELISA.

For determination of HLA restriction using inhibitory antibodies, T cells (10,000 cells/well) were incubated overnight with IE-1-expressing mini-LCLs (20,000 cells/well) in the presence of unlabeled purified antibodies specific for HLA-DR (clone L243, BioLegend), HLA-DQ (clone SPV-L3, AbD Serotec), or HLA-DP (clone B7/21, Abcam). For further determination of HLA restriction, T cells (10,000 cells/well) were incubated with control mini-LCLs and IE-1 mini-LCLs (20,000 cells/well) from a panel of 15 HLA-typed donors. After overnight incubation in V-bottom 96-well plates, supernatants were analyzed by ELISA. For determination of HLA restriction by transfection of HLA-encoding plasmids, 5 million DG75 cells were electroporated with a maximum of 20 μg of plasmid DNA (DRA- and DRB-coding sequences separately cloned into the pCMVcyto plasmid) in a Bio-Rad Gene Pulser (settings 230 V, 975 μF) in a 4-mm wide cuvette. Thereafter, cells were cultivated overnight, loaded with peptide, cocultivated together with T-cell clones over the following night (50,000 T cells and 50,000 transfected DG75 cells in 200 μL per V-bottom well), and supernatants were analyzed in IFN-γ ELISA.

## Results

**Figure 1:**
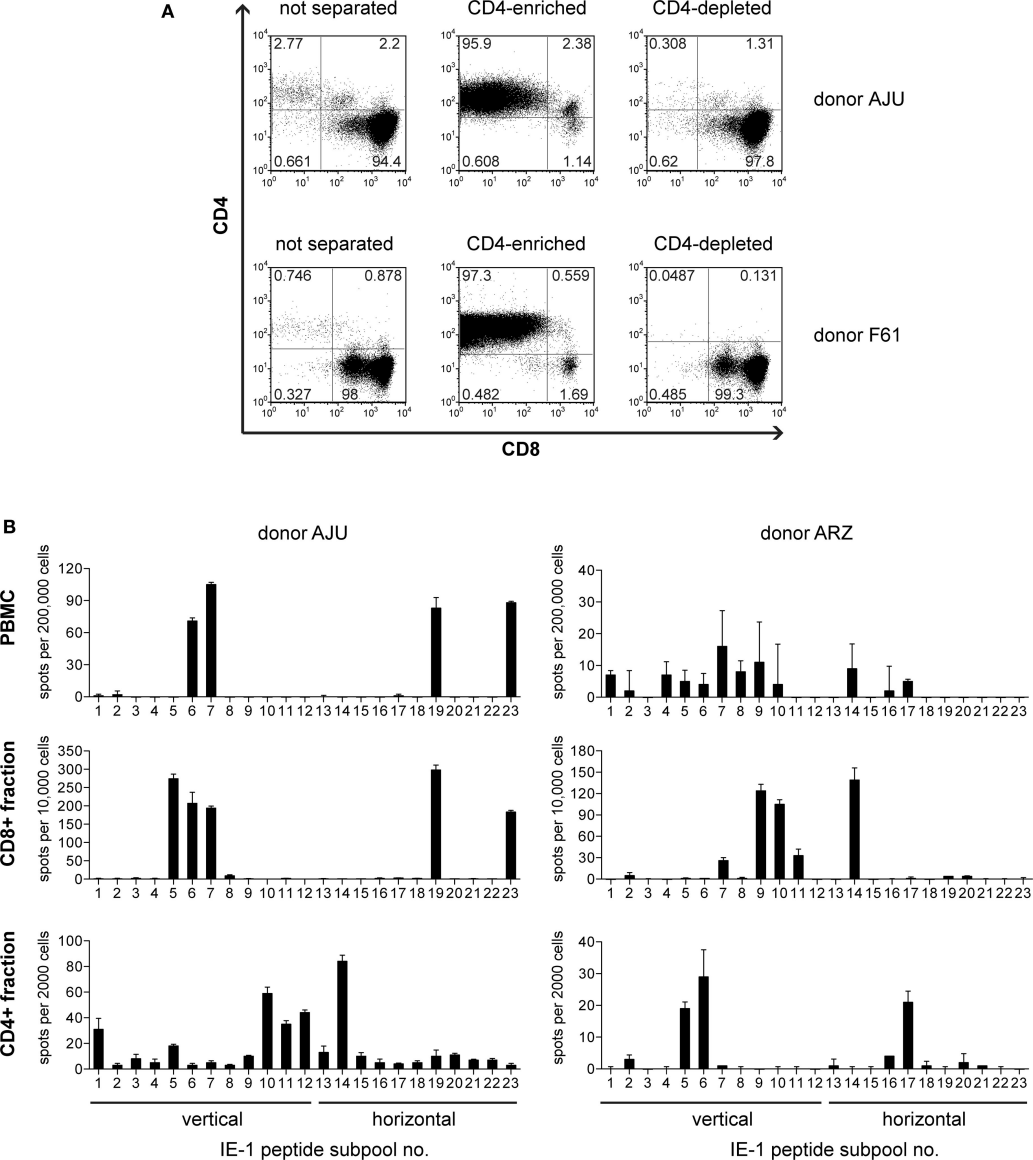
**Enrichment of IE-1-specific CD4 T cells**. **(A)** After 6–8 weeks of restimulation with the autologous IE-1 mini-LCL, T-cell cultures were immunomagnetically separated into CD4^+^ and CD4^−^ fractions, and the purity of these fractions was analyzed by flow cytometry. Results for two exemplary donors are shown. **(B)** PBMCs *ex vivo* and the separated CD4-enriched (CD4^+^) and CD4-depleted (CD8^+^) fractions of IE-1 mini-LCL-stimulated T-cell cultures were tested for their reactivity to IE-1 peptides in an IFN-γ ELISPOT assay. A peptide library covering the entire IE-1 protein sequence from HCMV strain AD169 plus some sequence variants from strains Toledo and TB40 was used for the stimulation of specific T cells in this assay. PBMCs were stimulated with peptide only, CD4^+^ and CD8^+^ fractions were stimulated with CD40-activated B cells loaded with peptides. Results are shown for two representative donors.

To enrich IE-1-specific T cells from peripheral blood from healthy HCMV carriers, we used a mini-EBV carrying the HCMV IE-1 gene under control of the SV40 early promoter (18). IE-1-expressing lymphoblastoid cell lines (mini-LCLs) were established from seven healthy HCMV IgG-positive adults, and PBMCs from these donors were restimulated with the autologous IE-1 mini-LCL for 6–8 weeks. The resulting polyclonal T-cell lines were heavily dominated by CD8^+^ T cells (18) but also contained a minor component of CD4^+^ T cells (3% on average). By immunomagnetic separation, we enriched this CD4^+^ component to an average of 97% (Figure 1A). To test whether these cells contained IE-1-specific CD4^+^ T cells, we stimulated them in an IFN-γ ELISPOT assay with a peptide library that represented the complete IE-1 sequence in the form of 15-mer peptides with an overlap of 11 amino acids, distributed to 23 different subpools. For each of the seven donors, the enriched CD4^+^ T cells recognized a subset of IE-1 peptide subpools, often others than were recognized by CD8^+^ (CD4-depleted) T cells from the same donor, and often ones that elicited no detectable reactivity from PBMC *ex vivo* (Figure 1B).

**Figure 2:**
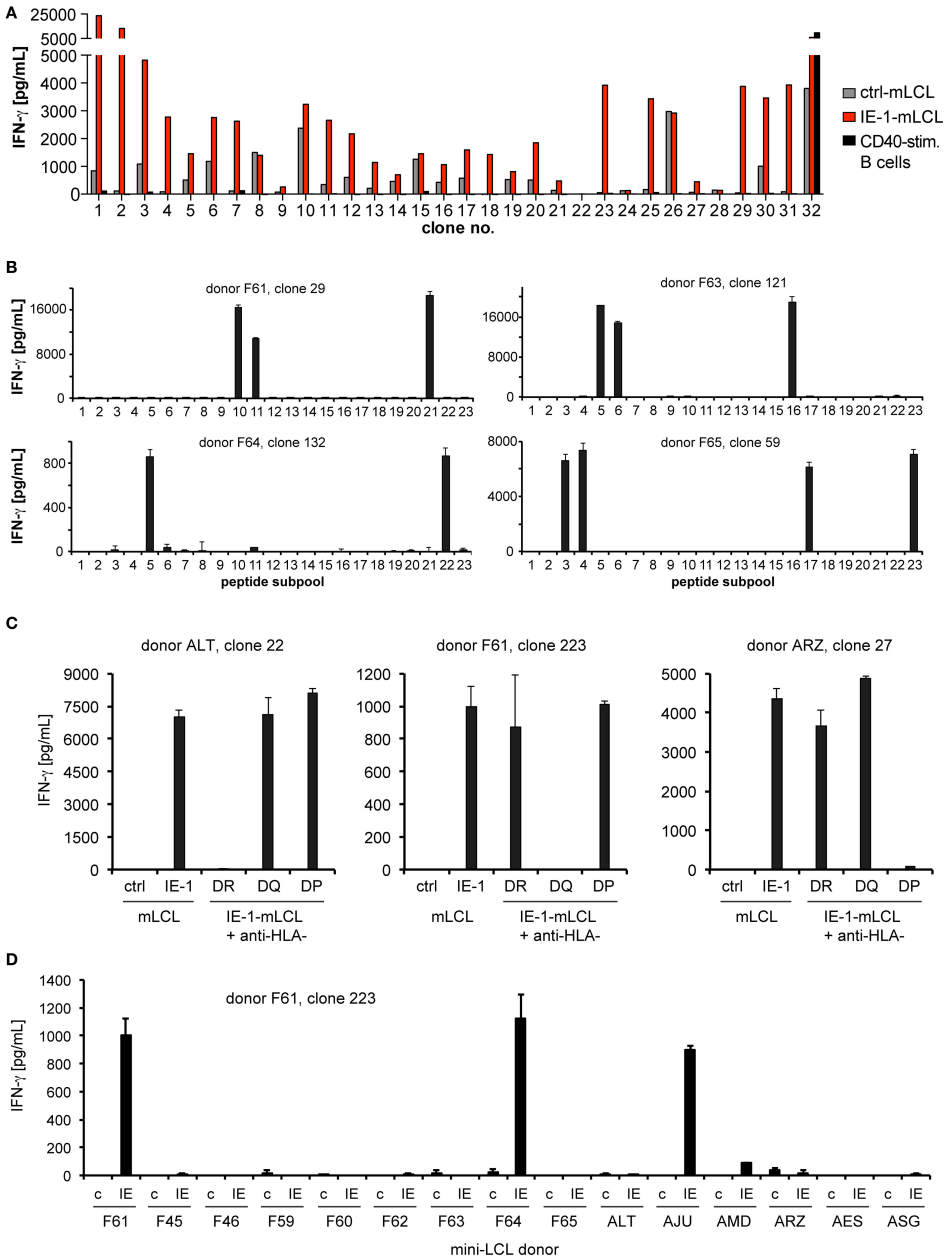
**Determination of specificity**. **(A)** In a first screen, recognition of endogenously processed IE-1 epitopes by CD4^+^ T-cell clones was determined by evaluating IFN-γ secretion (ELISA) in response to three different autologous target cell lines: an IE-1-expressing mini-LCL, an IE-1-negative mini-LCL (ctrl-mLCL). and non-infected, CD40-stimulated B cells. As an example, the panel shows the reactivity of the first 32 T-cell clones from donor F65. Only CD4 T-cell clones with a clear preference for the IE-1 mini-LCL were studied further. **(B)** IE-1 epitope specificity was determined by evaluating reactivity to the 23 subpools of the IE-1 peptide library. Peptides were loaded onto autologous mini-LCLs or CD40-stimulated B cells. The analysis of four representative T-cell clones is shown. **(C)** Restriction through the major subclasses of HLA class II was assessed by measuring blockade of IFN-γ secretion in the presence of specific antibodies to HLA-DR, -DQ, or -DP. Results are shown for three exemplary CD4^+^ T-cell clones. **(D)** Individual restricting HLA class II allotypes were deduced from the pattern of IFN-γ secretion in response to a panel of mini-LCLs from various HLA-typed donors, always including the IE-1-expressing mini-LCL (IE) and the IE-1-negative mini-LCL (“c” for control).

From these seven CD4-enriched T-cell cultures, we established T-cell clones by limiting dilution. At least 100 CD4^+^ T-cell clones from each donor were tested for their capability to recognize endogenously processed IE-1 epitopes, by comparing their recognition of autologous IE-1-expressing mini-LCLs, IE-1-negative mini-LCLs, and CD40-activated B cells. An example of such an analysis is shown in Figure 2A. Only T-cell clones with a clear IE-1-specific reactivity pattern (at least fivefold recognition of IE-1 mini-LCLs over controls) were selected for further study. A clear recognition pattern of IE-1 peptide subpools was obtained for a majority of these CD4^+^ T-cell clones, which allowed to determine their core peptide epitope with an 11- to 15-amino acid precision (Figure 2B). To narrow down their HLA restriction, we used inhibitory antibodies to HLA-DR, HLA-DQ, or HLA-DP (Figure 2C). HLA restriction was further specified by determining reactivity of T-cell clones to a panel of HLA-typed mini-LCLs. In the example shown in Figure 2D, HLA-DQ-restricted T-cell clone 223 from donor F61 recognized only the IE-1 mini-LCLs from three of 15 donors. The only HLA-DQA and DQB chains that were shared by these three donors but not others in the panel were DQA1*0201 and DQB1*0202. Thus, these were concluded to be the HLA class II chains presenting the epitope.

**Figure 3:**
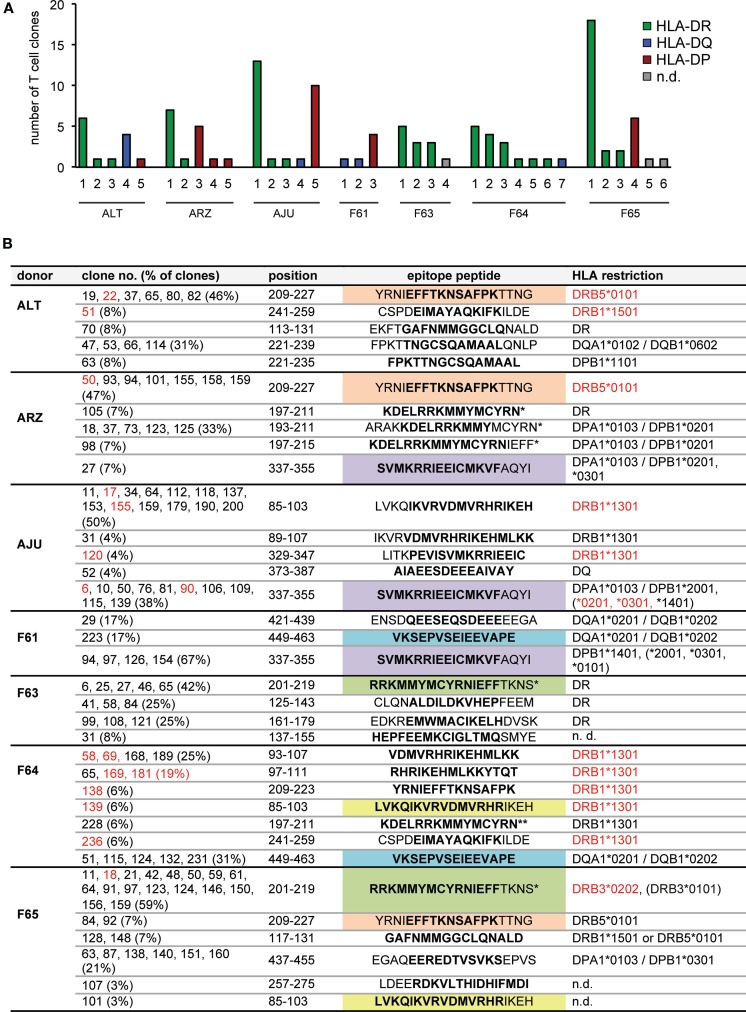
**CD4 T-cell epitopes in IE-1 and their HLA restrictions**. **(A)** Overview of the number and HLA restriction of different IE-1 epitope specificities per donor. **(B)** Full listing of epitopes and their HLA restrictions. Each T-cell clone recognized either a single 15-mer peptide or two adjacent peptides, covering 19 amino acids of the IE-1 sequence. Whenever two adjacent peptides were recognized with similar intensity (lower reactivity was greater than 50% of higher reactivity), their 11 amino acid overlap is shown in boldface; in other cases (lower reactivity between 10 and 50% of higher reactivity), the preferentially recognized 15-mer sequence is shown in boldface. Epitopes were considered distinct when their patterns of peptide recognition differed, even if they partially overlapped. Epitopes recognized by CD4 T-cell clones from more than one donor are highlighted in color. Entries in red text were verified in effector assays using transfection of single recombinant HLA molecules. Non-autologous HLA restrictions are in parentheses. An asterisk (*) indicates that both the strain AD169 sequence and the variant sequence from Toledo or TB40 were recognized. A double asterisk (**) indicates recognition of the AD169 sequence only.

These analyses of IE-1-specific CD4 T-cell clones showed that each of our seven donors harbored in their peripheral blood CD4 T cells specific for multiple IE-1 epitopes that were endogenously processed and presented. A median of five epitopes (range 3–7) per donor was identified (Figure 3A). Cumulatively, the CD4^+^ T cells from these seven donors recognized approximately 27 different epitopes from IE-1 (Figure 3B). T cells that recognized HLA-DR-restricted epitopes were found in six of seven donors, HLA-DQ-restricted epitopes in four donors, and HLA-DP-restricted epitopes in five. The most dominant single epitope-specific response – in terms of the number of clones that could be established – was HLA-DR-restricted in five donors, DP-restricted in one (F61), and DQ-restricted in one donor (F65). Several epitopes were shared between donors (Figure 3B).

The largest number of epitopes restricted through the same HLA allotype was found for DRB1*1301, which was carried by two of the donors and presented a total of nine different epitopes, according to their patterns of reactivity to peptide subpools. Five of these epitopes were located in region 85–111 of IE-1 and partially overlapping, which raises the possibility that some of these epitopes were not distinct, but recognized by different T-cell clones with different requirements for flanking residues outside the core epitope. The other four DRB1*1301-restricted epitopes were all non-overlapping and located in other regions of IE-1 (Figure 3B). Of note, two overlapping peptides (representing one or two epitopes) in the same 85–111 region were recently identified as being presented by DRB1*1301 (22). Determination of the precise number of distinct epitopes in this region will require additional analyses with potential minimal peptides at limiting dilution or by HLA/peptide tetramer staining (22).

Some largely overlapping epitopes were presented by more than one HLA allotype. A largely overlapping stretch in the 221–239 region was presented to T-cell clones from donor ALT either by HLA-DQ or by HLA-DP. Another epitope (CSPD**EIMAYAQKIFK**ILDE) was recognized by a DRB1*1501-restricted T-cell clone from donor ALT and by a DRB1*1301-restricted clone from donor F64.

HLA-DP-restricted T-cell clones tended to recognize peptides that were presented by more than one HLA-DP allotype. Three donors who carried one member of a related group of DPB1 alleles (*0301, *1401, or *2001) had T cells specific for the **SVMKRRIEEICMKVF**AQYI epitope, and such clones recognized this peptide not only on the autologous HLA-DP molecule but also on other HLA-DP allotypes inside or outside that group. DPB1 alleles *0301, *1401, and *2001 are closely related, with a maximum difference of two amino acid polymorphisms (31), which makes such a crossrecognition plausible.

Certain negative conclusions were also suggested by these results. Three donors (AJU, F61, and F64) were carriers of the DRB1*0701 allotype, and our results ruled out a restriction through this allotype for all clones from these donors. Therefore, HLA-DRB1*0701 appears less likely to present IE-1 epitopes, and to our knowledge no such epitopes have been described by others. However, DRB1*0701 is in linkage disequilibrium with DQA1*0201/DQB1*0202 as described (32), and thus carriers of this haplotype can instead target IE-1 through HLA-DQ-restricted CD4 T cells, as seen for two of our three donors with this haplotype (Table 1; Figure 3B).

**Figure 4:**
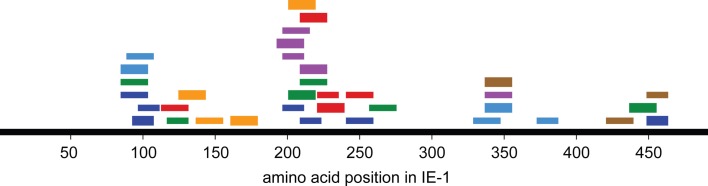
**Distribution of CD4 T-cell responses and epitopes within the IE-1 protein sequence**. Each bar represents an epitope-specific response in one donor. Different donors are represented by different colors. Thick bars represent an epitope-specific response of more than 20% of a donor’s T-cell clones, thin bars responses of 20% of clones or less.

An overview of the distribution of CD4 T-cell epitopes within the IE-1 protein sequence, color-coded by donor, is provided in Figure 4. Epitopes were not uniformly distributed, but were concentrated in certain regions of the protein. The concentration was the highest in region 193–239. Epitopes in this region were recognized by five of seven donors and had six distinct HLA restrictions, counting the promiscuous HLA-DP restriction of the **SVMKRRIEEICMKVF**AQYI epitope only once. Regions of high epitope density could be of particular interest for immunologic therapy or monitoring.

**Figure 5:**
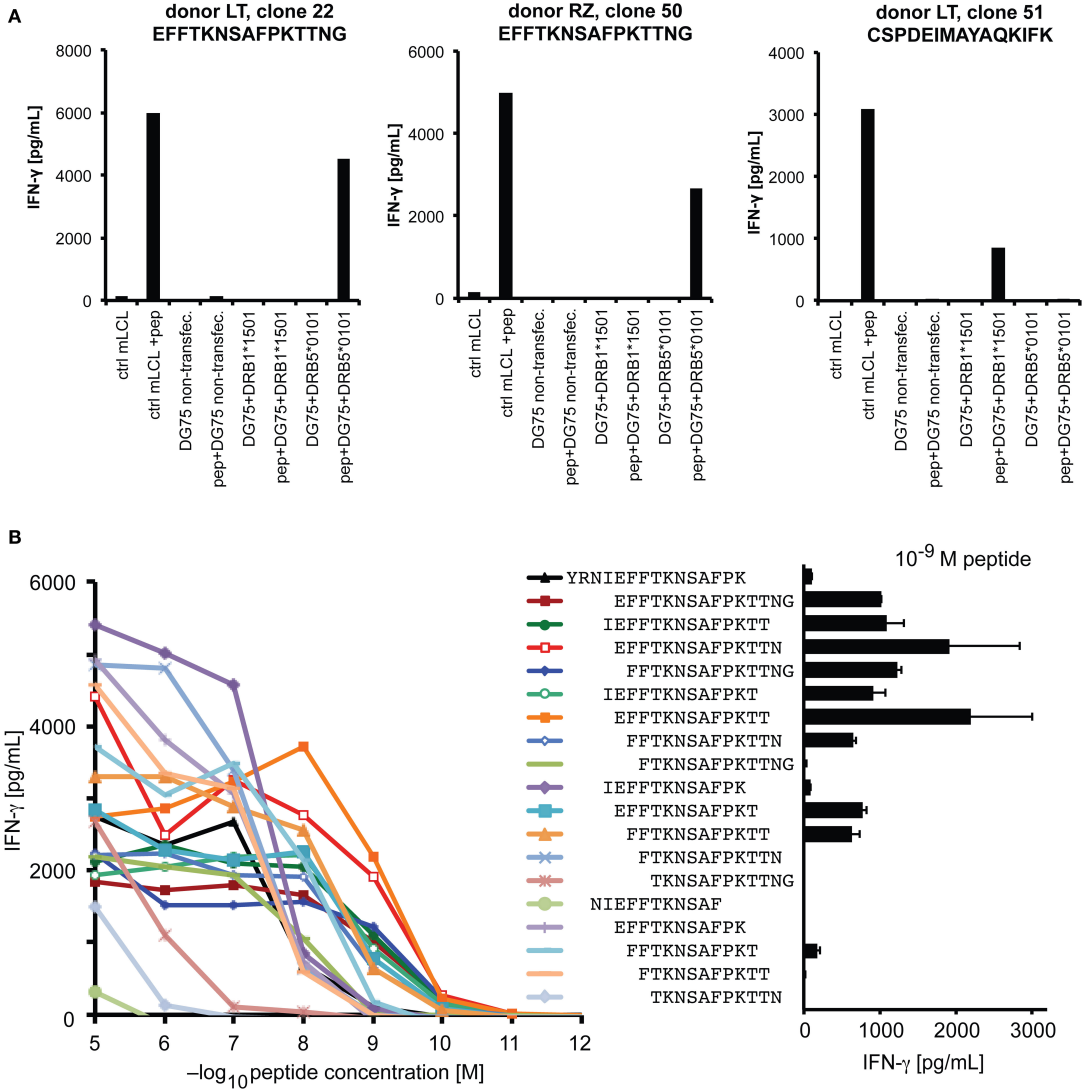
Analysis of an HLA-DRB5*0101-restricted epitope. **(A)** HLA restriction was molecularly defined by testing the reactivity of specific CD4 T-cell clones against DG75 cells that were transiently transfected with plasmids encoding the DRA chain and the indicated DRB chain, and transfectants were loaded with specific peptide where indicated. Recognition of peptide-loaded mini-LCL served as functional control. **(B)** Reactivity in response to titrated peptides representing various subsequences of the antigenic region. Peptides were loaded on autologous CD40-activated B cells, coincubated with clonal T cells overnight, and response was measured in an IFN-γ ELISA.

We performed a more detailed analysis of the HLA-DRB5*0101-restricted epitope YRNI**EFFTKNSAFPK**TTNG at position 209–227. The HLA-DRB1*1501/DRB5*0101 haplotype is of high frequency in persons of European and Asian descent (33, 34). Responses to this epitope were detected in all three donors with this haplotype, and in one of one additional haplotype carrier who was screened only for this epitope. Since the two DRB genes of this haplotype are in strong linkage disequilibrium, it was necessary to determine the restricting molecule by a molecular approach. An analysis of IFN-γ secretion by T-cell clones in response to peptide-loaded, single HLA-transfected DG75 cells (Figure 5A) showed that the 209–227 epitope was restricted through DRB5*0101, whereas the 241–259 epitope was restricted through DRB1*1501 (compare Figure 3B). We also determined the minimal epitope within the 209–227 region by analyzing the response to various subsequences of different length (Figure 5B). Since, at a low peptide concentration of 1nM, a 13-mer peptide EFFTKNSAFPKTT (position 213–225) was maximally recognized, whereas the two contained 12-mers elicited an equally reduced response and the central 11-mer only a weak response, we operationally defined the 13-mer to be the functional minimal epitope. This epitope, with its core motif FxxNxxxxK, is in relatively good accordance with a previously described motif of DRB1*0501 ligand peptides (35). So far, we were unable to detect specific IFN-γ-secreting CD4 T cells responsive to this epitope by ELISPOT *ex vivo* in peripheral blood, which suggests that their precursor frequency is below one in 50,000 cells.

**Figure 6:**
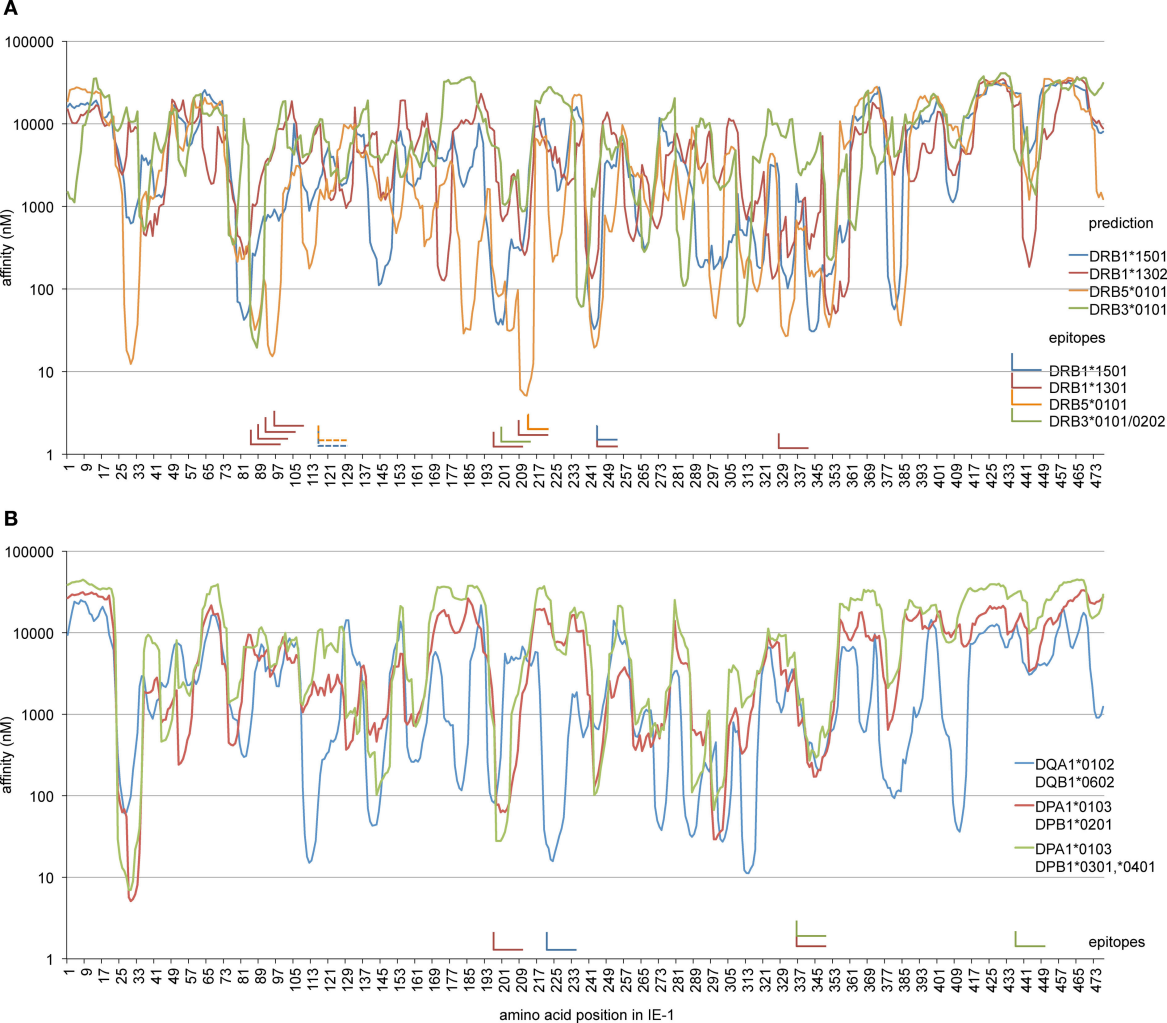
**Comparison of HLA binding predictions and confirmed epitopes**. The NetMHCII 2.2 algorithm was used to calculate predicted binding affinities (nM) along the IE-1 sequence for those allotypes that were restriction elements of epitopes identified in this study. Curves show predicted affinities in nanomolar for each 15-mer whose N terminus is located at the IE-1 amino acid position indicated on the *x*-axis. “L” shapes represent confirmed IE-1 epitopes. The horizontal arm of each “L” indicates the position of reference on the *x*-axis, the vertical arm points at the position of reference in the prediction curves. One epitope with two possible HLA restrictions is represented as a pair of dashed “L” shapes. **(A)** Prediction for HLA-DR allotypes and confirmed DR-restricted epitopes. Since prediction for HLA-DR*1301 was not available in NetMHCII, HLA-DR*1302 is shown instead. **(B)** Prediction for available HLA-DQ and DP allotypes that match epitopes identified in this study, and the position of corresponding DQ- or DP-restricted epitopes.

To analyze whether other epitopes identified here were in accordance with expectations regarding their HLA restriction, we used an advanced HLA class II binding prediction algorithm, NetMHCII 2.2 (36), to screen the IE-1 sequence for areas of predicted high binding affinity (Figure 6). The region of IE-1 with the highest predicted binding affinity to DRB5*0101 was precisely located at the position of the EFFTKNSAFPKTT epitope (Figure 6A). The DRB1*1501-restricted epitope 241–259 was located in one of four regions with predicted high binding affinity. Prediction for DRB1*1301 was not available in NetMHCII 2.2, so we analyzed prediction for DRB1*1302, but agreement with IE-1 epitopes was limited. The DRB3*0202/*0101-presented epitope at position 201–219 was not predicted by the algorithm for DRB3*0101. Available algorithms for specific HLA-DQ and HLA-DP heterodimers were in reasonably good agreement with most of our identified epitopes (Figure 6B).

## Discussion

Here, we show that IE-1-specific CD4 T cells can regularly be isolated from HCMV-positive donors of various HLA backgrounds. Although we studied a limited group of only seven donors, our robust detection of several specificities in each of these suggests that IE-1-specific CD4 T cells participate in antiviral T-cell responses in all or a majority of healthy carriers. CD4 T cells recognized IE-1 peptides presented by various HLA-DR, -DQ, or -DP allotypes, and each donor had T cells that were restricted through at least two of these three subclasses of HLA class II. A median of five (range 3–7) epitopes was recognized by T cells from each donor. Thus, IE-1-specific CD4 T-cell responses are widespread, diversified, and restricted by a wide range of different HLA class II allotypes. This observation provides arguments in favor of including IE-1 as a target antigen in immunotherapeutic approaches, such as multiepitope-specific adoptive T-cell transfer (37), with the intention to reconstitute specific CD4 as well as CD8 T cells. Our dataset will enable more comprehensive and precise analyses of the role of IE-1-specific CD4 T cells in immunocompromised patients, for example, by sensitive detection with HLA/peptide multimers (22).

Our results show that healthy HCMV-positive donors harbor IE-1-specific T cells even if they may not be easily detectable *ex vivo* with standard analytic methods. A seminal study identified proliferative responses to baculovirus-expressed IE-1 in about 60% of HCMV-positive donors (21). However, several subsequent studies have reported lower rates: IE-1-specific CD4 T cells were identified only in about one-third of HCMV carriers by measuring proliferation to IE-1-containing cell lysates (24) or by intracellular cytokine staining after peptide stimulation (9, 22). Other comprehensive studies employing peptide stimulation in connection with ELISPOT (25) or intracellular cytokine staining (26) did not identify IE-1-specific CD4 T cells in any of the donors studied or identified them only rarely (38). Like these researchers, we found it difficult to detect and quantify IE-1-specific CD4 T cells *ex vivo* by ELISPOT, presumably due to their low frequency, which prompted us to use specific expansion in cell culture in order to access these T cells for closer study. Since T-cell responses to other widespread human viruses that are well controlled by specific immunity can nonetheless elude *ex vivo* quantification but become detectable after expansion (39), it may not be surprising that the same is true for certain constituents of HCMV-specific T-cell immunity. Because T cells specific for a subset of antigens within a herpesvirus-specific T-cell repertoire may selectively home to specific tissues (40), low frequencies of IE-1-specific CD4 T cells in peripheral blood may not exclude an important role of these T cells in control of infection.

Studies on the functional role of IE-1-specific CD4 T cells are now easier than before, since with our present work a sufficient number of epitopes with different HLA restrictions has been identified to cover a majority of human HLA phenotypes. The chance that an HLA haplotype in a central European population contains at least one of the alleles DRB1*1301, DRB1*1501, DRB5*0101, DRB3*0101, DRB3*02, or DQB1*02 is 41%, resulting in a probability of 65% or higher that one or more of them appear in an HLA phenotype (41). This estimation still disregards HLA-DP, which will make an additional contribution to population coverage by known IE-1 epitopes, especially since we observed that T cells recognized the same epitope on a group of several mutually related HLA-DP allotypes. However, the HLA allotypes studied here are not only relevant for donors of European descent. HLA-DRB1*1501 and DRB5*0101 are among the most frequent HLA-DRB allotypes in East Asians as well as Caucasians (34, 42). HLA-DPB1*0201 is the second most frequent DPB1 allele both in East Asians and Caucasians (34, 43), and DRB3*0202 is present in Americans of African, European, and Asian descent at similar gene frequencies between 0.2 and 0.3 (42). Thus, the present set of epitopes will be useful for immunomonitoring or immunotherapy of patients of various ethnicities.

We identified approximately 27 different epitopes in total, but the exact number may be somewhat lower, since five discernable DRB1*1301-restricted epitope recognition patterns were derived from the region at amino acids 85–111 of IE-1, and some of these five functionally defined epitopes may not in fact correspond to distinct minimal epitopes. Moreover, two HLA-DPB1*0201-restricted specificities from donor ARZ recognized overlapping epitopes in the 197–211 region, and two DR-restricted epitopes from donors ALT and F65 overlap in the 113–131 region. Thus, our estimated lower limit of the number of distinct epitopes identified in our set of donors is 21. On the other hand, we cannot exclude the possibility that some of the functional epitopes identified in this study contain more than one minimal epitope. Since several epitope specificities were represented by only a single T-cell clone in our panel, and our analysis covered only a limited part of the human HLA class II repertoire, our analysis very likely underestimates the true diversity of the IE-1-specific CD4 T-cell response.

We have demonstrated that IE-1 epitopes can be restricted through all subclasses of HLA class II molecules, HLA-DR, -DQ and -DP, have enlarged the number of known HLA-DR-restricted epitopes, and have identified IE-1 epitopes restricted through HLA-DQ and HLA-DP for the first time. Some of the HLA-DR-restricted epitopes recognized by our T-cell clones have been described before. The 85–111 region of IE-1, where we have identified several DRB1*1301-restricted specificities, coincides with a previously identified DR13-restricted epitope in one donor (23), and in the same IE-1 region one or possibly two overlapping DRB1*1301-restricted epitopes recognized by another donor have been found (22). Our DRB5*0101-restricted epitope at position 213–225 coincides with a previously described epitope of the same restriction that was recognized by specific T cells in four of four carriers of this allele (22, 44), and the same region was dominantly targeted by CD4 T cells in a carrier of HLA-DR2 (21), which is compatible with its DRB5*0101 restriction. Thus, responses to these epitopes have so far been consistently found in several independent studies in all donors with the respective HLA phenotypes. These epitopes may tend to elicit responses that are particularly dominant among the IE-1-specific T-cell repertoire, which is indirectly corroborated by the relatively high number of corresponding T-cell clones that we obtained from our donors. Thus, our data, together with those of others, provide evidence for the existence of conserved immunodominance hierarchies among IE-1-specific CD4 T cells. Nonetheless, the CD4 T-cell repertoire responding to IE-1 is clearly more diversified and less influenced by immunodominance of individual epitopes than the CD8 T-cell repertoire, since IE-1-specific CD8 T cells in healthy HCMV carriers recognize, on average, fewer than two epitopes per donor (38).

While the role of IE-1-specific CD4 T cells in control of disease has been difficult to study due to their low frequency, there is convincing evidence that HCMV-specific CD4 T cells contribute to protection from disease after solid organ and stem cell transplantation (45–47). Moreover, strong therapeutic effects were shown in a study on adoptive transfer of predominantly CD4-positive HCMV-specific T cells after stem cell transplantation (8). In the murine CMV model, CD4 T cells are indispensable to control of viral replication in salivary glands (48, 49), and in the absence of CD8 T cells antiviral protection requires CD4 T cells (50).

Whether IE-1-specific T cells (CD8 or CD4) are of superior importance in control of infection has remained more controversial. For different human transplant situations, it was described that IE-1-specific T-cell responses, in contrast to T cells specific for the HCMV structural antigen pp65, are associated with control of HCMV disease (15, 16). However, others have observed an association of pp65-specific T cells with control of HCMV (51, 52), and therapeutic transfer of pp65-specific T cells was associated with reduction or clearance of manifest infection (5, 6). Since HCMV interferes with antigen presentation to T cells in an HLA class I allotype-specific manner (18, 53), individual epitopes from the same antigen may strongly differ in the efficacy of their presentation to CD8 T cells (18, 19). Thus, which HCMV proteins assume the role of protective antigens may depend on the HLA allotypes that are available in a particular HCMV carrier. Information of this kind has so far been lacking for IE-1-specific CD4 T cells, but identification of their target epitopes makes it now possible to undertake precise analyses of their role in combating infection and disease and to explore their function in adoptive T-cell therapy.

## Conflict of Interest Statement

The authors declare that the research was conducted in the absence of any commercial or financial relationships that could be construed as a potential conflict of interest.

## References

[1] Boeckh M Geballe AP. *Cytomegalovirus*: pathogen, paradigm, and puzzle. J Clin Invest (2011) 121:1673–80. 10.1172/JCI45449 21659716 PMC3083799

[2] Crough T Khanna R. Immunobiology of human *Cytomegalovirus*: from bench to bedside. Clin Microbiol Rev (2009) 22:76–98. 10.1128/CMR.00034-08 19136435 PMC2620639

[3] Quinnan GVJ Kirmani N Rook AH Manischewitz JF Jackson L Moreschi G Cytotoxic T cells in *Cytomegalovirus* infection: HLA-restricted T-lymphocyte and non-T-lymphocyte cytotoxic responses correlate with recovery from *Cytomegalovirus* infection in bone-marrow-transplant recipients. N Engl J Med (1982) 307:7–13. 10.1056/NEJM198207013070102 6281647

[4] Kenneson A Cannon MJ. Review and meta-analysis of the epidemiology of congenital *Cytomegalovirus* (CMV) infection. Rev Med Virol (2007) 17:253–76. 10.1002/rmv.535 17579921

[5] Walter EA Greenberg PD Gilbert MJ Finch RJ Watanabe KS Thomas ED Reconstitution of cellular immunity against *Cytomegalovirus* in recipients of allogeneic bone marrow by transfer of T-cell clones from the donor. N Engl J Med (1995) 333:1038–44. 10.1056/NEJM199510193331603 7675046

[6] Cobbold M Khan N Pourgheysari B Tauro S McDonald D Osman H Adoptive transfer of *Cytomegalovirus*-specific CTL to stem cell transplant patients after selection by HLA-peptide tetramers. J Exp Med (2005) 202:379–86. 10.1084/jem.20040613 16061727 PMC2213070

[7] Schmitt A Tonn T Busch DH Grigoleit GU Einsele H Odendahl M Adoptive transfer and selective reconstitution of streptamer-selected *Cytomegalovirus*-specific CD8+ T cells leads to virus clearance in patients after allogeneic peripheral blood stem cell transplantation. Transfusion (2011) 51:591–9. 10.1111/j.1537-2995.2010.02940.x 21133926

[8] Einsele H Roosnek E Rufer N Sinzger C Riegler S Loffler J Infusion of *Cytomegalovirus* (CMV)-specific T cells for the treatment of CMV infection not responding to antiviral chemotherapy. Blood (2002) 99:3916–22. 10.1182/blood.V99.11.3916 12010789

[9] Sylwester AW Mitchell BL Edgar JB Taormina C Pelte C Ruchti F Broadly targeted human *Cytomegalovirus*-specific CD4+ and CD8+ T cells dominate the memory compartments of exposed subjects. J Exp Med (2005) 202:673–85. 10.1084/jem.20050882 16147978 PMC2212883

[10] Kern F Surel IP Faulhaber N Frommel C Schneider-Mergener J Schonemann C Target structures of the CD8(+)-T-cell response to human *Cytomegalovirus*: the 72-kilodalton major immediate-early protein revisited. J Virol (1999) 73:8179–84. 10482568 10.1128/jvi.73.10.8179-8184.1999 PMC112835

[11] Wills MR Carmichael AJ Mynard K Jin X Weekes MP Plachter B The human cytotoxic T-lymphocyte (CTL) response to *Cytomegalovirus* is dominated by structural protein pp65: frequency, specificity, and T-cell receptor usage of pp65-specific CTL. J Virol (1996) 70:7569–79. 8892876 10.1128/jvi.70.11.7569-7579.1996 PMC190825

[12] Jonjic S del Val M Keil GM Reddehase MJ Koszinowski UH. A nonstructural viral protein expressed by a recombinant *Vaccinia virus* protects against lethal *Cytomegalovirus* infection. J Virol (1988) 62:1653–8. 2833615 10.1128/jvi.62.5.1653-1658.1988 PMC253194

[13] Reddehase MJ Mutter W Munch K Buhring HJ Koszinowski UH. CD8-positive T lymphocytes specific for murine *Cytomegalovirus* immediate-early antigens mediate protective immunity. J Virol (1987) 61:3102–8. 3041033 10.1128/jvi.61.10.3102-3108.1987 PMC255886

[14] Simon CO Holtappels R Tervo HM Bohm V Daubner T Oehrlein-Karpi SA CD8 T cells control *Cytomegalovirus* latency by epitope-specific sensing of transcriptional reactivation. J Virol (2006) 80:10436–56. 10.1128/JVI.01248-06 16928768 PMC1641801

[15] Bunde T Kirchner A Hoffmeister B Habedank D Hetzer R Cherepnev G Protection from *Cytomegalovirus* after transplantation is correlated with immediate early 1-specific CD8 T cells. J Exp Med (2005) 201:1031–6. 10.1084/jem.20042384 15795239 PMC2213133

[16] Sacre K Nguyen S Deback C Carcelain G Vernant JP Leblond V Expansion of human *Cytomegalovirus* (HCMV) immediate-early 1-specific CD8+ T cells and control of HCMV replication after allogeneic stem cell transplantation. J Virol (2008) 82:10143–52. 10.1128/JVI.00688-08 18684826 PMC2566250

[17] Manley TJ Luy L Jones T Boeckh M Mutimer H Riddell SR. Immune evasion proteins of human *Cytomegalovirus* do not prevent a diverse CD8+ cytotoxic T-cell response in natural infection. Blood (2004) 104:1075–82. 10.1182/blood-2003-06-1937 15039282

[18] Ameres S Mautner J Schlott F Neuenhahn M Busch DH Plachter B Presentation of an immunodominant immediate-early CD8+ T cell epitope resists human *Cytomegalovirus* immunoevasion. PLoS Pathog (2013) 9:e1003383. 10.1371/journal.ppat.1003383 23717207 PMC3662661

[19] Ameres S Besold K Plachter B Moosmann A. CD8 T cell-evasive functions of human *Cytomegalovirus* display pervasive MHC allele specificity, complementarity, and cooperativity. J Immunol (2014) 192:5894–905. 10.4049/jimmunol.1302281 24808364

[20] Borysiewicz LK Hickling JK Graham S Sinclair J Cranage MP Smith GL Human *Cytomegalovirus*-specific cytotoxic T cells. Relative frequency of stage-specific CTL recognizing the 72-kD immediate early protein and glycoprotein B expressed by recombinant *Vaccinia viruses*. J Exp Med (1988) 168:919–31. 10.1084/jem.168.3.919 2844952 PMC2189029

[21] Alp NJ Allport TD Van Zanten J Rodgers B Sissons JG Borysiewicz LK. Fine specificity of cellular immune responses in humans to human *Cytomegalovirus* immediate-early 1 protein. J Virol (1991) 65:4812–20. 1714519 10.1128/jvi.65.9.4812-4820.1991 PMC248939

[22] Braendstrup P Mortensen BK Justesen S Osterby T Rasmussen M Hansen AM Identification and HLA-tetramer-validation of human CD4+ and CD8+ T cell responses against HCMV proteins IE1 and IE2. PLoS One (2014) 9:e94892. 10.1371/journal.pone.0094892 24760079 PMC3997423

[23] Davignon JL Castanie P Yorke JA Gautier N Clement D Davrinche C. Anti-human *Cytomegalovirus* activity of cytokines produced by CD4+ T-cell clones specifically activated by IE1 peptides in vitro. J Virol (1996) 70:2162–9. 8642638 10.1128/jvi.70.4.2162-2169.1996 PMC190054

[24] Davignon JL Clement D Alriquet J Michelson S Davrinche C. Analysis of the proliferative T cell response to human *Cytomegalovirus* major immediate-early protein (IE1): phenotype, frequency and variability. Scand J Immunol (1995) 41:247–55. 10.1111/j.1365-3083.1995.tb03560.x 7871384

[25] Nastke MD Herrgen L Walter S Wernet D Rammensee HG Stevanovic S. Major contribution of codominant CD8 and CD4 T cell epitopes to the human *Cytomegalovirus*-specific T cell repertoire. Cell Mol Life Sci (2005) 62:77–86. 10.1007/s00018-004-4363-x 15619009 PMC11924560

[26] Slezak SL Bettinotti M Selleri S Adams S Marincola FM Stroncek DF. CMV pp65 and IE-1 T cell epitopes recognized by healthy subjects. J Transl Med (2007) 5:17. 10.1186/1479-5876-5-17 17391521 PMC1851947

[27] Moosmann A Khan N Cobbold M Zentz C Delecluse HJ Hollweck G B cells immortalized by a mini-*Epstein-Barr virus* encoding a foreign antigen efficiently reactivate specific cytotoxic T cells. Blood (2002) 100:1755–64. 12176897

[28] Wiesner M Zentz C Mayr C Wimmer R Hammerschmidt W Zeidler R Conditional immortalization of human B cells by CD40 ligation. PLoS One (2008) 3:e1464. 10.1371/journal.pone.0001464 18213373 PMC2180193

[29] Kern F Bunde T Faulhaber N Kiecker F Khatamzas E Rudawski IM *Cytomegalovirus* (CMV) phosphoprotein 65 makes a large contribution to shaping the T cell repertoire in CMV-exposed individuals. J Infect Dis (2002) 185:1709–16. 10.1086/340637 12085315

[30] Wiesner M Zentz C Hammer MH Cobbold M Kern F Kolb HJ Selection of CMV-specific CD8+ and CD4+ T cells by mini-EBV-transformed B cell lines. Eur J Immunol (2005) 35:2110–21. 10.1002/eji.200425936 15971271

[31] Zino E Frumento G Marktel S Sormani MP Ficara F Di Terlizzi S A T-cell epitope encoded by a subset of HLA-DPB1 alleles determines nonpermissive mismatches for hematologic stem cell transplantation. Blood (2004) 103:1417–24. 10.1182/blood-2003-04-1279 14576061

[32] Klitz W Maiers M Spellman S Baxter-Lowe LA Schmeckpeper B Williams TM New HLA haplotype frequency reference standards: high-resolution and large sample typing of HLA DR-DQ haplotypes in a sample of European Americans. Tissue Antigens (2003) 62:296–307. 10.1034/j.1399-0039.2003.00103.x 12974796

[33] Schmidt AH Baier D Solloch UV Stahr A Cereb N Wassmuth R Estimation of high-resolution HLA-A, -B, -C, -DRB1 allele and haplotype frequencies based on 8862 German stem cell donors and implications for strategic donor registry planning. Hum Immunol (2009) 70:895–902. 10.1016/j.humimm.2009.08.006 19683023

[34] Trachtenberg E Vinson M Hayes E Hsu YM Houtchens K Erlich H HLA class I (A, B, C) and class II (DRB1, DQA1, DQB1, DPB1) alleles and haplotypes in the Han from southern China. Tissue Antigens (2007) 70:455–63. 10.1111/j.1399-0039.2007.00932.x 17900288

[35] Vogt AB Kropshofer H Kalbacher H Kalbus M Rammensee HG Coligan JE Ligand motifs of HLA-DRB5*0101 and DRB1*1501 molecules delineated from self-peptides. J Immunol (1994) 153:1665–73. 7519208

[36] Nielsen M Lund O. NN-align. An artificial neural network-based alignment algorithm for MHC class II peptide binding prediction. BMC Bioinformatics (2009) 10:296. 10.1186/1471-2105-10-296 19765293 PMC2753847

[37] Papadopoulou A Gerdemann U Katari UL Tzannou I Liu H Martinez C Activity of broad-spectrum T cells as treatment for AdV, EBV, CMV, BKV, and HHV6 infections after HSCT. Sci Transl Med (2014) 6:242ra83 10.1126/scitranslmed.3008825 PMC4181611 24964991

[38] Khan N Best D Bruton R Nayak L Rickinson AB Moss PA. T cell recognition patterns of immunodominant *Cytomegalovirus* antigens in primary and persistent infection. J Immunol (2007) 178:4455–65. 10.4049/jimmunol.178.7.4455 17372003

[39] Martin LK Schub A Dillinger S Moosmann A. Specific CD8(+) T cells recognize human herpesvirus 6B. Eur J Immunol (2012) 42:2901–12. 10.1002/eji.201242439 22886850

[40] Hislop AD Kuo M Drake-Lee AB Akbar AN Bergler W Hammerschmitt N Tonsillar homing of *Epstein-Barr virus*-specific CD8+ T cells and the virus-host balance. J Clin Invest (2005) 115:2546–55. 10.1172/JCI24810 16110323 PMC1187932

[41] Knipper AJ Hakenberg P Enczmann J Kuhrober A Kiesel U Kogler G HLA-DRB1,3,4,5 and -DQB1 allele frequencies and HLA-DR/DQ linkage disequilibrium of 231 German caucasoid patients and their corresponding 821 potential unrelated stem cell transplants. Hum Immunol (2000) 61:605–14. 10.1016/S0198-8859(00)00114-2 10825589

[42] Maiers M Gragert L Klitz W High-resolution HLA alleles and haplotypes in the United States population. Hum Immunol (2007) 68:779–88. 10.1016/j.humimm.2007.04.005 17869653

[43] Hollenbach JA Madbouly A Gragert L Vierra-Green C Flesch S Spellman S A combined DPA1~DPB1 amino acid epitope is the primary unit of selection on the HLA-DP heterodimer. Immunogenetics (2012) 64:559–69. 10.1007/s00251-012-0615-3 22526601 PMC3395342

[44] Braendstrup P Justesen S Osterbye T Nielsen LL Mallone R Vindelov L MHC class II tetramers made from isolated recombinant alpha and beta chains refolded with affinity-tagged peptides. PLoS One (2013) 8:e73648. 10.1371/journal.pone.0073648 24023895 PMC3759463

[45] Gabanti E Bruno F Lilleri D Fornara C Zelini P Cane I Human *Cytomegalovirus* (HCMV)-specific CD4+ and CD8+ T cells are both required for prevention of HCMV disease in seropositive solid-organ transplant recipients. PLoS One (2014) 9:e106044. 10.1371/journal.pone.0106044 25166270 PMC4148399

[46] Gamadia LE Remmerswaal EB Weel JF Bemelman F van Lier RA Ten Berge IJ. Primary immune responses to human CMV: a critical role for IFN-gamma-producing CD4+ T cells in protection against CMV disease. Blood (2003) 101:2686–92. 10.1182/blood-2002-08-2502 12411292

[47] Hebart H Daginik S Stevanovic S Grigoleit U Dobler A Baur M Sensitive detection of human *Cytomegalovirus* peptide-specific cytotoxic T-lymphocyte responses by interferon-gamma-enzyme-linked immunospot assay and flow cytometry in healthy individuals and in patients after allogeneic stem cell transplantation. Blood (2002) 99:3830–7. 10.1182/blood.V99.10.3830 11986243

[48] Jonjic S Mutter W Weiland F Reddehase MJ Koszinowski UH. Site-restricted persistent *Cytomegalovirus* infection after selective long-term depletion of CD4+ T lymphocytes. J Exp Med (1989) 169:1199–212. 10.1084/jem.169.4.1199 2564415 PMC2189231

[49] Walton SM Mandaric S Torti N Zimmermann A Hengel H Oxenius A. Absence of cross-presenting cells in the salivary gland and viral immune evasion confine *Cytomegalovirus* immune control to effector CD4 T cells. PLoS Pathog (2011) 7:e1002214. 10.1371/journal.ppat.1002214 21901102 PMC3161985

[50] Jonjic S Pavic I Lucin P Rukavina D Koszinowski UH. Efficacious control of *Cytomegalovirus* infection after long-term depletion of CD8+ T lymphocytes. J Virol (1990) 64:5457–64. 1976821 10.1128/jvi.64.11.5457-5464.1990 PMC248597

[51] Cwynarski K Ainsworth J Cobbold M Wagner S Mahendra P Apperley J Direct visualization of *Cytomegalovirus*-specific T-cell reconstitution after allogeneic stem cell transplantation. Blood (2001) 97:1232–40. 10.1182/blood.V97.5.1232 11222365

[52] Gratama JW Brooimans RA van der Holt B Sintnicolaas K van Doornum G Niesters HG Monitoring *Cytomegalovirus* IE-1 and pp65-specific CD4+ and CD8+ T-cell responses after allogeneic stem cell transplantation may identify patients at risk for recurrent CMV reactivations. Cytometry B Clin Cytom (2008) 74:211–20. 10.1002/cyto.b.20420 18454493

[53] Schust DJ Tortorella D Seebach J Phan C Ploegh HL. Trophoblast class I major histocompatibility complex (MHC) products are resistant to rapid degradation imposed by the human *Cytomegalovirus* (HCMV) gene products US2 and US11. J Exp Med (1998) 188:497–503. 10.1084/jem.188.3.497 9687527 PMC2212475

